# Interaction of Cucurbit[7]uril With Protease Substrates: Application to Nanosecond Time-Resolved Fluorescence Assays

**DOI:** 10.3389/fchem.2020.00806

**Published:** 2020-09-10

**Authors:** Andreas Hennig, Werner M. Nau

**Affiliations:** ^1^Department of Life Sciences and Chemistry, Jacobs University Bremen gGmbH, Bremen, Germany; ^2^Institute of Chemistry of New Materials, School of Biology/Chemistry, Universität Osnabrück, Osnabrück, Germany; ^3^Center of Cellular Nanoanalytics (CellNanOs), Universität Osnabrück, Osnabrück, Germany

**Keywords:** cucurbiturils, enzyme assays, proteases, time-resolved fluorescence (TRF), peptides

## Abstract

We report the use of the macrocyclic host cucurbit[7]uril (CB7) as a supramolecular additive in nanosecond time-resolved fluorescence (Nano-TRF) assays for proteases to enhance the discrimination of substrates and products and, thereby, the sensitivity. A peptide substrate was labeled with 2,3-diazabicyclo[2.2.2]oct-2-ene (DBO) as a long-lived (>300 ns) fluorescent probe and 3-nitrotyrosine was established as a non-fluorescent fluorescence resonance energy transfer (FRET) acceptor that acts as a “dark quencher.” The substrate was cleaved by the model proteases trypsin and chymotrypsin and the effects of the addition of CB7 to the enzyme assay mixture were investigated in detail using UV/VIS absorption as well as steady-state and time-resolved fluorescence spectroscopy. This also allowed us to identify the DBO and nitrotyrosine residues as preferential binding sites for CB7 and suggested a hairpin conformation of the peptide, in which the guanidinium side chain of an arginine residue is additionally bound to a vacant ureido rim of one of the CB7 hosts.

**Graphical Abstract:**
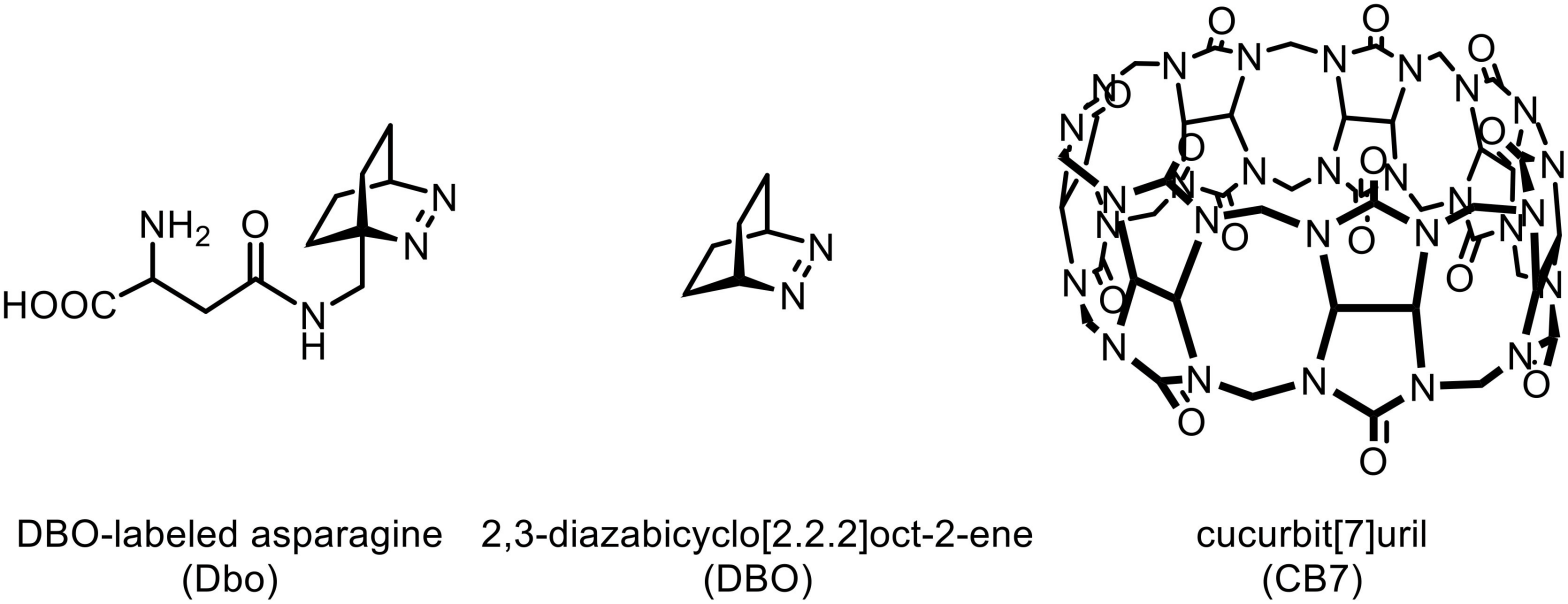
Chemical structures of Dbo, DBO, and CB7.

## Introduction

Time-resolved fluorescence (TRF) detection has become an indispensable tool in biochemical research (Liu and Hennig, in press; Hemmilä and Webb, 1997; Steinkamp and Karst, 2004), which has been applied to enzyme assays (Karvinen et al., 2004; Sadler et al., 2004; Terai et al., 2006; Vuojola et al., 2013; Hewitt and Butler, 2018), fluoroimmunoassays (Hildebrandt et al., 2005; Charbonnière et al., 2006; Geißler et al., 2010), and assays involving oligonucleotides (Johansson et al., 2004; Wu et al., 2013). Commonly, lanthanide chelates have been employed as luminescent probes with lifetimes in the millisecond time range (Bünzli, 2010). Since typical fluorescence lifetimes are <10 ns, such exceedingly long probe lifetimes allow to record the probe emission after a short delay time during which the short-lived background emission has already significantly decayed (Hemmilä and Laitala, 2005). Such a time-gated measurement affords an efficient reduction of short-lived background emission and an increased sensitivity (Hemmilä and Webb, 1997; Steinkamp and Karst, 2004) However, lanthanide chelates suffer from several drawbacks, i.e., a large size, a cationic center, and hydrophobic ligands, which may interfere with the biomolecular enzyme-substrate recognition. Thus, organic fluorescent probes with contrasting properties are often desirable for complementary applications (Turconi et al., 2001; Gribbon and Sewing, 2003; Kainmüller et al., 2005; Hennig et al., 2006, 2007b; Sahoo et al., 2007a).

With the advent of fast-switching electric circuits, shorter lifetimes in the range of 100 ns to several microseconds are nowadays also accessible for TRF detection, and ruthenium complexes have been suggested as suitable probes, which are, however, similarly problematic (Bannwarth et al., 1988; Hennig and Zeckert, 2000; Yun et al., 2003; Kainmüller et al., 2005; Kainmüller and Bannwarth, 2006; Clima et al., 2007; Martí et al., 2007a,b; Kramer et al., 2008; Kolpashchikov, 2010; Guo et al., 2011). Further, pyrene has been explored for its utility in TRF assays with nanosecond lifetimes (Yang et al., 2005; Martí et al., 2006, 2007a,b; Conlon et al., 2008; Guo et al., 2011), but large hydrophobic, aromatic surfaces, such as that of pyrene, are well-known to interfere with biomolecular recognition (Daugherty and Gellman, 1999; Sahoo et al., 2007a). As an appealing alternative, we have introduced 2,3-diazabicyclo[2.2.2]oct-2-ene labeled asparagine (Dbo), which exhibits an unquenched lifetime of ca. 330 ns in water (Hudgins et al., 2002; Nau and Wang, 2002). Application of Dbo to TRF assays for proteases, kinases, and phosphatases demonstrated for the first time the practical feasibility of this concept, for which we have coined the name nanosecond time-resolved fluorescence (Nano-TRF) assays (Hennig et al., 2006, 2007b; Sahoo et al., 2007a).

From previous work, it was known that the parent molecule 2,3-diazabicyclo[2.2.2]oct-2-ene (DBO) forms a strong supramolecular host-guest inclusion complex with the macrocyclic host cucurbit[7]uril (CB7) (Márquez and Nau, 2001; Marquez et al., 2004; Mohanty and Nau, 2004). The extremely low polarizability of the CB7 cavity, which lies between that of perfluorohexane and the gas phase, leads—according to the Strickler-Berg relation—to a prolonged radiative decay rate of fluorescent dyes resulting in an increased fluorescence lifetime of the CB7/DBO complex of up to 1,050 ns (Strickler and Berg, 1962; Márquez and Nau, 2001; Marquez et al., 2004; Mohanty and Nau, 2004; Nau and Mohanty, 2005). In addition, other desirable effects of CB7 complexation on fluorescent dyes have been observed, e.g., an enhanced stability, increased brightness, deaggregation, and a reduced intermolecular quenching (Márquez et al., 2003; Mohanty and Nau, 2005; Nau and Mohanty, 2005; Koner and Nau, 2007). It has consequently been proposed that the advantageous effects of CB7 on fluorescent dyes could be exploited for the construction of improved enzyme assays (Marquez et al., 2004). Herein, we explore the feasibility of this concept by applying CB7 to a Nano-TRF protease assay in which we introduce 3-nitrotyrosine as a “dark,” that is, non-fluorescent quencher of the DBO chromophore. Proteases were selected due to their importance as targets in drug discovery (Hennig and Zeckert, 2000; Hildebrandt et al., 2005; Clima et al., 2007; Hennig et al., 2007b; Sahoo et al., 2007a; Vuojola et al., 2013). We provide a detailed photophysical characterization of this novel assay and elucidate the interactions of CB7 with the substrate. Finally, we demonstrate that the lifetime-enhancing effect of CB7 presents a powerful enhancement strategy for Nano-TRF assays.

## Materials and Methods

### Peptide Substrates and Enzymes

DBO-labeled asparagine was synthesized according to a literature procedure (Hudgins et al., 2002). The peptide was prepared by Biosyntan GmbH (Berlin, Germany) and obtained in >95% purity. Trypsin (from bovine pancreas, 2,500 U/mg) and chymotrypsin (CT, from bovine pancreas, 1,500 U/mg) were from AppliChem (Darmstadt, Germany). We used a 67 mm phosphate buffer (pH 7) for CT and a 112 mm borate buffer (pH 8) for trypsin. CB7 was synthesized according to the literature procedure (Kim et al., 2000; Day et al., 2001; Marquez et al., 2004).

### Fluorescence Spectroscopy

Absorption measurements of enzyme and peptide stock solutions were performed with a Varian Cary 4000 spectrophotometer. The following extinction coefficients were used to derive the concentrations of the enzymes: ε_280_ = 33,600 m^−1^cm^−1^ for trypsin (Labouesse and Gervais, 1967) and ε_280_ = 50,000 m^−1^cm^−1^ for CT (Martin and Marini, 1967). For 3-nitrotyrosine we determined an extinction coefficient of ε_378_ = 2,260 ± 50 m^−1^cm^−1^ (the isosbestic point in titrations with CB7), which we used for measuring the peptide concentrations. Fluorescence lifetimes were determined by time-correlated single photon counting (FLS920, Edinburgh Instruments) with a Picoquant picosecond pulsed diode laser (λ_exc_ = 373 nm, λ_em_ = 450 nm, ca. 50 ps pulse width) for excitation. The reported lifetimes were recovered by tail-fitting with the instrument-specific software. The goodness-of-fit was judged by a reduced χ^2^ of <1.1 and a random distribution of the weighted residuals around zero, if not otherwise specified. Kinetic traces were collected with a Varian Eclipse spectrofluorometer, equipped with a thermostatted cell holder (λ_exc_ = 365 nm, λ_em_ = 450 nm).

### Kinetic Assays

1 millimolar stock solutions of the peptide were prepared in water and stored at +4°C. For the assays, an appropriate amount was diluted with the respective buffer solution and the sample was allowed to equilibrate for at least 10 min in a thermostatted cell holder at 25.0 ± 0.1°C. Enzyme stock solutions were freshly prepared on a daily basis in 1 mM HCl and small aliquots of the enzyme stock solution were added to the reaction mixture in 67 mM sodium phosphate buffer, pH 7.0 (for chymotrypsin) or 112 mM sodium borate buffer, pH 8.0 (for trypsin). It was ensured that the small aliquot of 1 mM HCl has no influence on the pH of the reaction mixture.

Nano-TRF assays were carried out in black 384-well microplates (Corning NBS) using 50 μl final sample volume with an LF 402 NanoScan FI microplate reader (IOM, Berlin, Germany). An external nitrogen laser (MNL 200, Laser Technik Berlin, Germany) was coupled by glass fibers to a dye laser module, which had a maximum dye emission at 365 nm. The emission was detected at 450 nm with a gate time of 2 μs.

## Results and Discussion

### Choice and Characterization of the Probe-Quencher Pair

The natural amino acids tryptophan and tyrosine have been exploited as intrinsic contact-based quenchers of the DBO chromophore in the previously reported Nano-TRF assays (Liu and Hennig, in press; Hennig et al., 2006, 2007b; Sahoo et al., 2007a). The use of these natural quenching sites is, however, not feasible for the current approach, because CB7 acts not only as a lifetime-enhancing agent but also as an efficient “protection shield” against contact-based quenching (Márquez et al., 2003; Marquez et al., 2004; Nau et al., 2003). Both effects are inherently related to the formation of supramolecular host-guest inclusion complexes, where the DBO chomophore, as a guest, is deeply immersed inside the cavity of CB7, the host. We therefore needed to resort to conventional fluorescence resonance energy transfer (FRET) to construct substrates for proteases (Matayoshi et al., 1990; Gershkovich and Kholodovych, 1996). FRET does not require an intimate probe-quencher contact but acts through space (Förster, 1948; Stryer and Haugland, 1967; Selvin and Hearst, 1994) and also through the walls of the macrocyclic host (Zhang et al., 2019).

**Figure 1:**
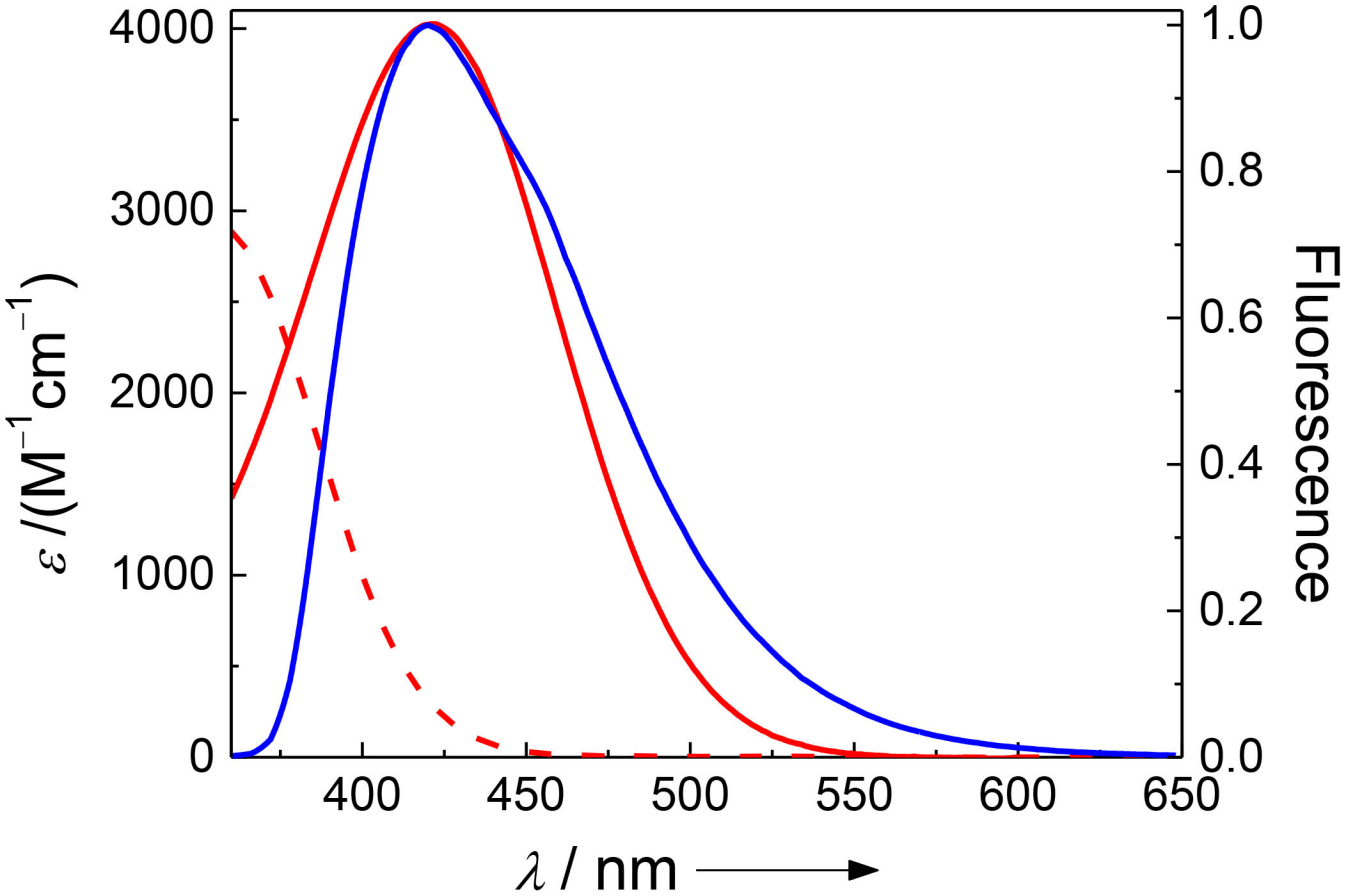
Spectral overlap between the pH-independent emission of Dbo (blue) and the absorption of 3-nitrotyrosine (red) at pH 2 (dashed line) and pH 8 (solid line).

While FRET with Dbo as an acceptor (note that Dbo designates the entire amino acid, while DBO designates the neat chromophore, see structure chart) is being used to assess peptide folding rates and distance distributions (Sahoo et al., 2006, 2007b; Jacob et al., 2013, 2018; Norouzy et al., 2015), the use of Dbo as a donor has not been reported so far, and we thus sought for an efficient energy acceptor. We selected 3-nitrotyrosine (NO_2_)Tyr, for several reasons: First, the absorption of (NO_2_)Tyr excellently overlaps with the emission of Dbo (Figure 1) in the biologically relevant pH range, ensuring efficient quenching. Second, (NO_2_)Tyr is a non-fluorescent dark quencher, which by-passes complications owing to residual acceptor fluorescence (Grüninger-Leitch et al., 2002; Johansson et al., 2004). Third, (NO_2_)Tyr has a very low affinity toward CB7, which allows the selective complexation of Dbo in the presence of the acceptor (*vide infra*). And fourth, (NO_2_)Tyr is a relatively small and not too hydrophobic molecule compared to many fluorescent dyes. The latter has the added advantage that a small residue is less prone to interfere with the enzyme-substrate recognition than a larger molecule (Gershkovich and Kholodovych, 1996; Hennig et al., 2006; Christopher et al., 2011). In other words, while we needed to sacrifice the possibility of employing an intrinsic quencher like tryptophan or tyrosine, we selected a quencher that has a similarly low propensity to interfere with the enzyme activity as the previously employed quenchers (Hennig et al., 2006, 2007b; Sahoo et al., 2007a) but that exhibits a different quenching mechanism; the latter allowed, as an added advantage, that other beneficial effects of CB7 encapsulation, namely its lifetime-enhancing effect on Dbo, could be fully exploited, while maintaining effective quenching.

The absorption spectra of (NO_2_)Tyr were measured in the pH range from 2.0 to 9.0 (Table 1) to assess the expected assay performance of the Dbo/(NO_2_)Tyr FRET pair. The absorption spectral properties of (NO_2_)Tyr compare well with literature data (ε_420_ = 4189 m^−1^cm^−1^ for pH > 8 and ε_365_ = 2761 m^−1^cm^−1^ for pH < 6.8) (Fisher and Naughton, 2003), and the reported p*K*_a_ of (NO_2_)Tyr of 6.8 was precisely reproduced (6.79 ± 0.02). The Förster distance, *R*_0_, was calculated from the donor emission spectrum and the acceptor absorption spectra according to the established methods, using an orientation factor of 2/3 and a refractive index of 1.333 (Lakowicz, 2006; Sahoo et al., 2006; Sloniec et al., 2013). This provided an estimate of the energy transfer efficiency *E* for two typical lengths (10 and 15 Å) of a terminally probe-quencher-labeled protease substrate (Table 1). Details on the determination of *R*_0_ and the energy transfer efficiency have been previously reported and are included as Supporting Information (Birks and Georghiou, 1968). Quenching efficiencies >95% in the relevant pH range (pH 7–8) predict a minimum 20-fold increase in fluorescence intensity for the Dbo/(NO_2_)Tyr probe/quencher pair in protease assays, which represents a considerable improvement to our previously reported assays with tryptophan and tyrosine as quenchers (Hennig et al., 2007b), and which is comparable to the best available protease assays based on FRET (Matayoshi et al., 1990; Gershkovich and Kholodovych, 1996; Christopher et al., 2011).

**Table 1:** Dependence of the Förster critical radius *R*_0_, the absorption maximum λ_max_, and the extinction coefficient ε of the Dbo/3-nitrotyrosine FRET pair on pH.^a^

**pH**	***R*_0_/Å**	**λ_max_ (nm)**	**ε (m^−1^cm^−1^)**	***E* (10 Å)^b^**	***E* (15 Å)^c^**
2.0	17.9	355	2,930	97.0%	74.2%
3.0	18.0	355	2,930	97.1%	74.9%
4.0	18.1	356	2,880	97.2%	75.5%
5.0	18.4	356	2,840	97.5%	77.3%
6.0	21.0	359	2,620	98.8%	88.3%
7.0	24.3	416	2,760	99.5%	94.8%
8.0	26.6	422	4,030	99.7%	96.9%
9.0	27.1	424	4,360	99.7%	97.2%

a *Error is <5% (largest around the pK_a_ of nitrotyrosine)*.

b *Length of a typical 6-mer peptide (Sahoo et al., 2006)*.

c *Length of a typical 10-mer peptide (Sahoo et al., 2006)*.

### Effects of Donor/Acceptor Diffusion on FRET

For a long-lived fluorescent probe like Dbo in connection with FRET, it is necessary to take the effects of mutual probe-acceptor diffusion into consideration (Yokota and Tanimoto, 1967; Birks and Georghiou, 1968; Steinberg and Katchalski, 1968; Thomas et al., 1978). Therefore, we determined the fluorescence lifetimes of the parent chromophore DBO and of the CB7/DBO complex in the presence of 0–100 μm (NO_2_)Tyr at pH 8. The decay traces were in all cases monoexponential, as judged by a reduced χ^2^ close to 1.0 and a random distribution of the weighted residuals around zero. A tentative Stern-Volmer analysis revealed apparent intermolecular quenching rate constants of 8.0 × 10^9^ m^−1^s^−1^ for DBO and 4.7 × 10^9^ m^−1^s^−1^ for the CB7/DBO complex.

Such monoexponential decay traces can, however, not be expected *a priori* for FRET-based quenching. The theory on the effects of diffusion on FRET is complex, but several limiting cases have been described (Yokota and Tanimoto, 1967; Birks and Georghiou, 1968; Steinberg and Katchalski, 1968; Thomas et al., 1978). Stryer and co-workers introduced for two freely diffusing molecules in solution the parameter *D*τ_0_/*s*^2^, in which *D* is the sum of the diffusion coefficients of donor *D*_d_ and acceptor *D*_a_, τ_0_ is the fluorescence lifetime of the donor in absence of FRET, and *s* is the mean distance between donor and acceptor [*s* = 0.5(*c*_A_*N*_A_)^1/3^, with *c*_A_ being the acceptor concentration and *N*_A_ the Avogadro number] (Thomas et al., 1978). A long fluorescence lifetime and a rapid diffusion lead to *D*τ_0_/*s*^2^ >> 1. This allows the acceptor molecules to reach an equilibrium distribution during the lifetime and monoexponential fluorescence lifetimes are expected for such a case. If *D*τ_0_/*s*^2^ << 1, the donor molecules can be regarded as static and a multiexponential distribution of fluorescence lifetimes results, which reflects the uneven distribution of acceptor molecules during the fluorescence lifetime. The former case applies to extremely long excited-state lifetimes, e.g., those of lanthanide chelates, and the latter case best describes common fluorescent dyes with lifetimes <25 ns.

Medium-range lifetimes such as that of Dbo, for which *D*τ_0_/*s*^2^ is in the range of 1.1–6.2 require, however, a more elaborate treatment. Birks and Georghiou (1968) suggested the use of the model of Yokota and Tanimoto (1967) for the limiting case that (2*D*τ_0_)^1/2^ > 3*R*_0_. This criterion was met for all our donor-acceptor combinations, and we accordingly performed simulations with this model. The simulated decay traces could be fitted with a monoexponential decay function as well and followed the Stern-Volmer equation, as did our experimental traces. The simulated quenching rate constants of 4.9 × 10^9^ m^−1^s^−1^ for DBO as donor and 3.7 × 10^9^ m^−1^s^−1^ for the CB7/DBO complex as a donor are in satisfactory agreement with the experimental values (*vide supra*).

Most important to note, the experimental as well as the simulated values lie distinctly above the theoretical rate constants for intermolecular diffusion-limited reactions, *k*_diff_, in water (4.7 × 10^9^ m^−1^s^−1^ for the DBO/(NO_2_)Tyr encounter and 2.9 × 10^9^ m^−1^s^−1^ for the encounter of (NO_2_)Tyr with the larger CB7/DBO complex at 20°C) (Lapidus et al., 2000; Lee et al., 2003; Huang et al., 2004; Sahoo et al., 2006,^1^. This conclusively demonstrates that the quenching proceeds primarily by FRET and that there is at best a small contribution of contact-based quenching. Interestingly, ruthenium-based complexes have been used as FRET donors as well as acceptors (Bannwarth et al., 1988; Hennig and Zeckert, 2000; Yun et al., 2003; Kainmüller et al., 2005; Kainmüller and Bannwarth, 2006; Clima et al., 2007; Martí et al., 2007a,b; Kramer et al., 2008; Kolpashchikov, 2010; Guo et al., 2011), but the effects of diffusion in this time range has yet not been characterized in the context of bioassays. The present study might therefore provide a basis for the further application of biolabels in the microsecond time range.

### Design of Protease Substrate and Expected Assay Performance

We designed a substrate for trypsin and chymotrypsin, two well-accepted model proteases (Fischer et al., 2002; Hedstrom, 2002; Park et al., 2002). The peptide sequence for the protease substrate was derived from known (Smyth, 1967) recognition motifs; appending a (NO_2_)Tyr *N*-terminal and a Dbo *C*-terminal to the recognition sequence afforded the desired FRET substrate: H-(NO_2_)Tyr-Gly-Ser-Gly-Phe-Arg-Gly-Dbo-NH_2_. As expected, the substrate is cleaved by chymotrypsin and trypsin, and the fluorescence intensity increased 15 and 35 times, respectively, as expected from the quenching behavior of the neat fluorophores.

The increase in fluorescence intensity is accompanied by an increase in fluorescence lifetime to 325 ns after proteolytic cleavage, which resembles the lifetime of previously reported values for an unquenched Dbo and which is sufficiently long to allow Nano-TRF detection (Hudgins et al., 2002; Hennig et al., 2006, 2007b; Sahoo et al., 2007a). The fluorescence lifetime after proteolytic cleavage can be practically regarded constant (within 10% error) up to a substrate concentration of 50 μm. Knowledge of the upper concentration range is, however, essential for a reliable determination of enzyme kinetic parameters or inhibition constants (Grüninger-Leitch et al., 2002). Alternatively, the lifetime dependence on the (NO_2_)Tyr concentration can be corrected for, if necessary, according to the intermolecular diffusion model (*vide supra*) (Yokota and Tanimoto, 1967).

For the uncleaved peptide, however, an unexpected result was obtained (Birks and Georghiou, 1968; Thomas et al., 1978). We expected a monoexponential fluorescence lifetime, because *D*τ_0_/*s*^2^ is ca. 10–100 for the uncleaved peptide (Wang et al., 2003,^2^) which should allow for a better description by the rapid diffusion model (Thomas et al., 1978). In contrast, we observed a pronounced multiexponential decay, which could not be satisfactorily fitted (reduced χ^2^ >> 1 and a non-random distribution of the weighted residuals around zero in case of all attempted fittings). We therefore presume the formation of several relatively long-lived low-energy conformations of the peptide, each contributing with a different fluorescence lifetime. This is not unlikely in view of the possibility of ground-state complexes between the electron-rich phenylalanine group and the electron-poor nitrotyrosine (Yaron et al., 1979; Packard et al., 1996; Johansson and Cook, 2003); interestingly, dissociation rate constants in the relevant range for tryptophan and oxazine dyes in peptides have, in fact, been detected by single-molecule spectroscopy (Neuweiler et al., 2003). Despite the difficulties to reliably fit and interpret the fluorescence lifetime of the uncleaved peptide, it is important to note that the lifetime is nonetheless sufficiently short (ca. 5 ns, reduced χ^2^ = 10) to be efficiently suppressed by Nano-TRF detection.

### Substrate Binding Studies With CB7

Cucurbit[*n*]urils constitute a class of macrocyclic oligomers composed of *n* glycoluril units, which are supramolecular host molecules with outstanding molecular recognition properties that have been applied as stimuli-responsive systems, e.g., in catalysis, bioanalysis, drug delivery, or in polymeric and nano-sized materials (Bhasikuttan et al., 2011; Masson et al., 2012; Isaacs, 2014; Kaifer, 2014; Assaf and Nau, 2015; Barrow et al., 2015; Cong et al., 2016; van Dun et al., 2017; Kim, 2020). The most studied homologs with *n* = 6, 7, and 8 are known to bind to various amino acids, peptides, and proteins with affinities in the range of 10^3^-10^9^ M^−1^, and the molecular recognition properties indicated a preference for hydrophobic and cationic amino acid residues (Urbach and Ramalingam, 2011; Gamal-Eldin and Macartney, 2013; Logsdon and Urbach, 2013; Lee et al., 2015a,b; Smith et al., 2015; Li et al., 2016; Webber et al., 2016; Hirani et al., 2018). In our own work, we have exploited the interactions of cucurbiturils with amino acids, peptides, and proteins for the sensing of enzyme activity, membrane transport, and chirality (Hennig et al., 2007a; Bailey et al., 2008; Nau et al., 2009; Dsouza et al., 2012; Biedermann and Nau, 2014; Ghale et al., 2014; Schnurr et al., 2015; Nilam et al., 2017; Barba-Bon et al., 2019; Liu et al., 2019; Biedermann et al., 2020). It was therefore considered to be very likely that CB7 will bind to several sites of the polyfunctional substrate H-(NO_2_)Tyr-Gly-Ser-Gly-Phe-Arg-Gly-Dbo-NH_2_ with different affinity, in particular because DBO is also known to strongly bind with CB7 (Marquez et al., 2004).

**Figure 2:**
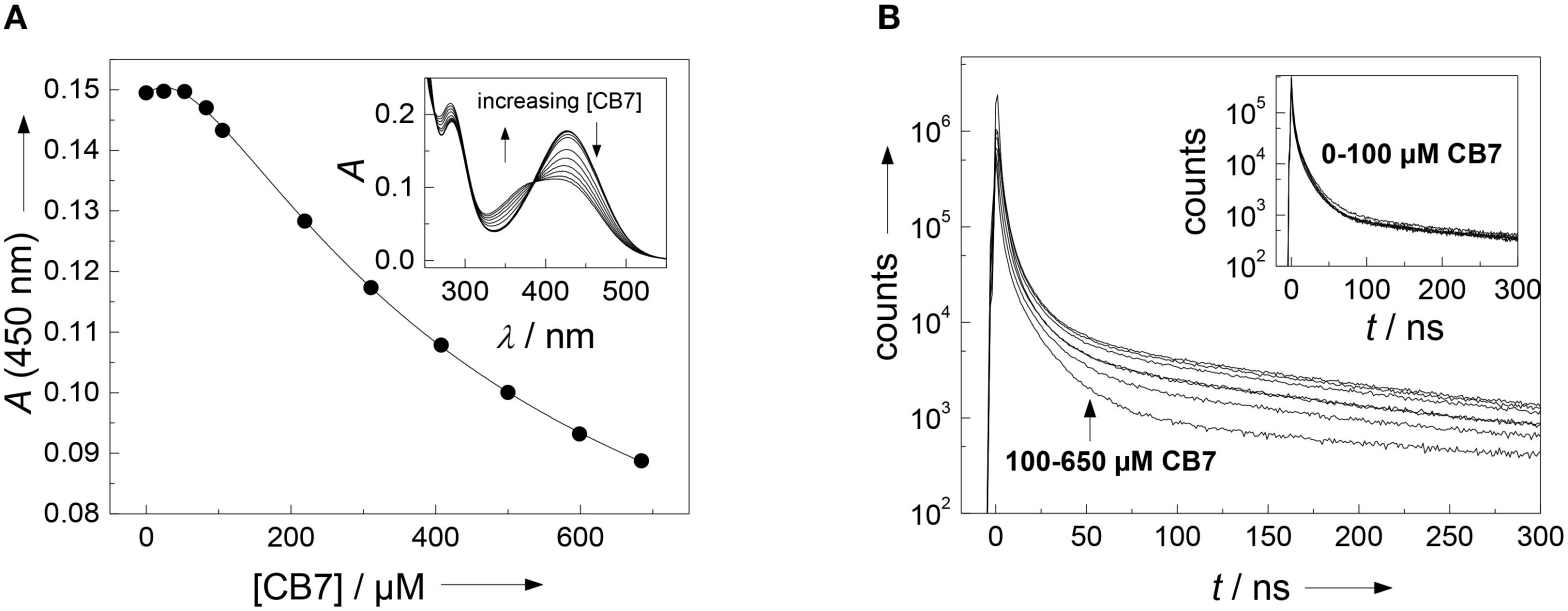
**(A)** Determination of the binding constant between the FRET acceptor 3-nitrotyrosine and CB7. Shown is the titration curve of a 40 μm peptide solution with varying amounts of CB7 in borate buffer, pH 8. The solid line indicates the fitting according to a 2:1 host-guest binding model. The inset shows the respective UV absorption spectra. **(B)** Dependence of fluorescence decay traces of the peptide H-(NO_2_)Tyr-Gly-Ser-Gly-Phe-Arg-Gly-Dbo-NH_2_ (30 μm) on CB7 concentration. The experiments were performed in 67 mm phosphate buffer, pH 7.

It often presents a major challenge to dissect the contribution of each amino acid side chain to the overall binding, but we will show that the properties of the present substrate allow the combined use of several independent analytical techniques. Straightforward to interpret are the changes on the absorption spectrum in the range of the (NO_2_)Tyr absorption (Figure 2A). The 430 nm absorption band of (NO_2_)Tyr decreases upon addition of increasing concentrations of CB7, while the absorption below 385 nm increases with an apparent isosbestic point at 385 nm^3^. The initial lag phase, in which the absorption does not change upon CB7 addition immediately suggests a 2:1 host-guest binding (Bakirci and Nau, 2005). The weaker binding constant of (2,200 ± 500) m ^−1^ is associated with encapsulation of the (NO_2_)Tyr side chain into the CB7 cavity, because the change in the (NO_2_)Tyr absorption is most pronounced at high CB7 concentrations. At low CB7 concentrations, no change in the (NO_2_)Tyr absorption is observed suggesting that the stronger binding constant of (1.2 ± 0.7) × 10^4^ m^−1^ does not involve the (NO_2_)Tyr side chain but must take place at a different position of the substrate (*vide infra*). Unfortunately, there is yet not much available data for a comprehensive interpretation, but a binding constant of 2,200 m^−1^ with CB7 is reasonable in view of a binding constant of 2,400 m^−1^ for Trp-OMe with CB7 (Hennig et al., 2007c). The formation of a 2:1 complex is further supported by time-correlated single photon counting (TCSPC) experiments. At low CB7 concentrations (up to ca. 100 μm), there is virtually no change in the fluorescence decay traces (see inset of Figure 2B), but at higher CB7 concentrations a long-lived component evolves (see Figure 2B). A quantitative analysis was not attempted at this point, because of the complex multiexponential decay (*vide supra*), but it is obvious that the decrease in the (NO_2_)Tyr absorption band centered at 430 nm (compare Figure 2A), which is the maximum of the Dbo emission, will lead to a less efficient FRET and, thus, to a prolonged fluorescence lifetime.

Since it was not clear, which part of the intact peptide is responsible for the higher binding constant, titrations with the cleaved peptide fragments were performed. Chymotrypsin preferentially hydrolyzes peptide bonds *N*-terminally to hydrophobic residues, which results in the peptide fragments H-(NO_2_)Tyr-Gly-Ser-Gly-Phe-OH and H-Arg-Gly-Dbo-NH_2_. Trypsin preferentially hydrolyzes peptide bonds *N*-terminally to cationic residues, which results in the peptide fragments H-(NO_2_)Tyr-Gly-Ser-Gly-Phe-Arg-OH and H-Gly-Dbo-NH_2_. Addition of small amounts of CB7 after cleavage to either mixture led to a biexponential decay with one component of 325 ns and a second component with 950 ns. The contribution as expressed by the preexponential factor of the longer-lived component increased with increasing concentrations of CB7, while the shorter-lived decreased, which clearly suggests the formation of a Dbo/CB7 inclusion complex.

**Figure 3:**
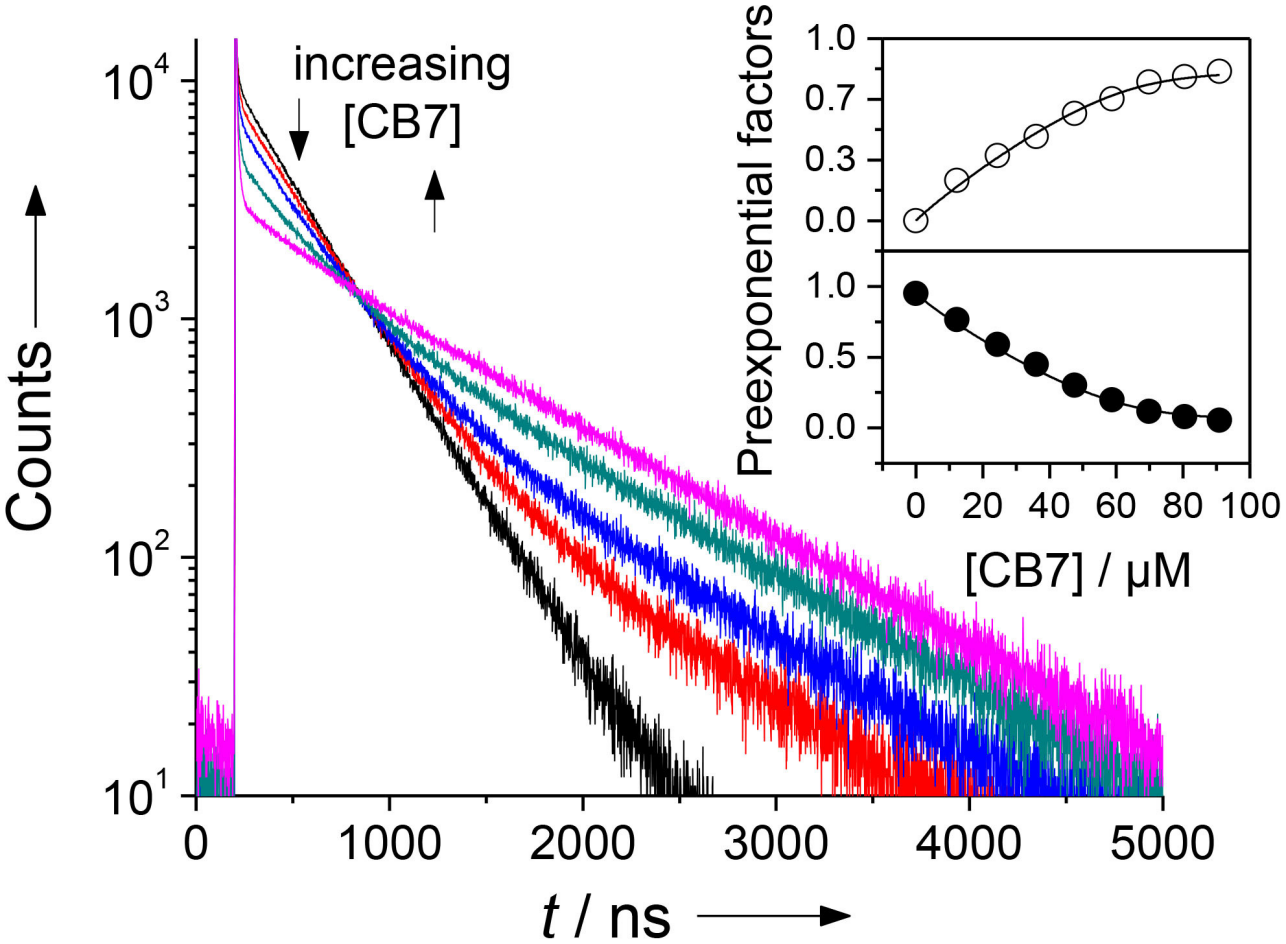
Fluorescence lifetime enhancing effect upon addition of increasing amounts of CB7 to an assay mixture after complete cleavage of 30 μm H-(NO_2_)Tyr-Gly-Ser-Gly-Phe-Arg-Gly-Dbo-NH_2_ by chymotrypsin in phosphate buffer, pH 7. The different traces represent CB7 concentrations of 0, 12, 24, 36, and 70 μm. The sharp peak immediately after the laser pulse is attributed to a minor fluorescent impurity. The inset shows the dependence of the normalized preexponential factors on the CB7 concentration (upper panel, open circles: long-live component with 950 ns; lower panel, filled circles: short-lived component with 325 ns fluorescence lifetime), which provided a suitable titration curve. The solid lines were obtained by fitting according to a 1:1 host-guest binding model.

The titrations (inset of Figure 3) could be excellently described according to a 1:1 host-guest binding model and revealed a binding constant of (1.9 ± 0.5) × 10^4^ m^−1^. This binding constant of the cleaved peptide fragments is in excellent agreement with the independently determined first binding constant from the 2:1 fitting of the UV absorption titration for the uncleaved peptide. Based on the evident encapsulation of Dbo after cleavage and because the cleavage site is remote from the complexation site, it can, thus, be inferred that Dbo is also preferentially complexed in the uncleaved peptide. Although the complexation of Dbo in the uncleaved peptide had no effect on the fluorescence decay traces, this was not deteriorating for the desired application, for which solely the effect on the fluorescence lifetime after cleavage is essential. In the Discussion section, we will provide a comprehensive model to explain the peculiar effects supported by enzyme kinetic measurements (*vide infra*).

### Enzyme Kinetics With Trypsin

**Figure 4:**
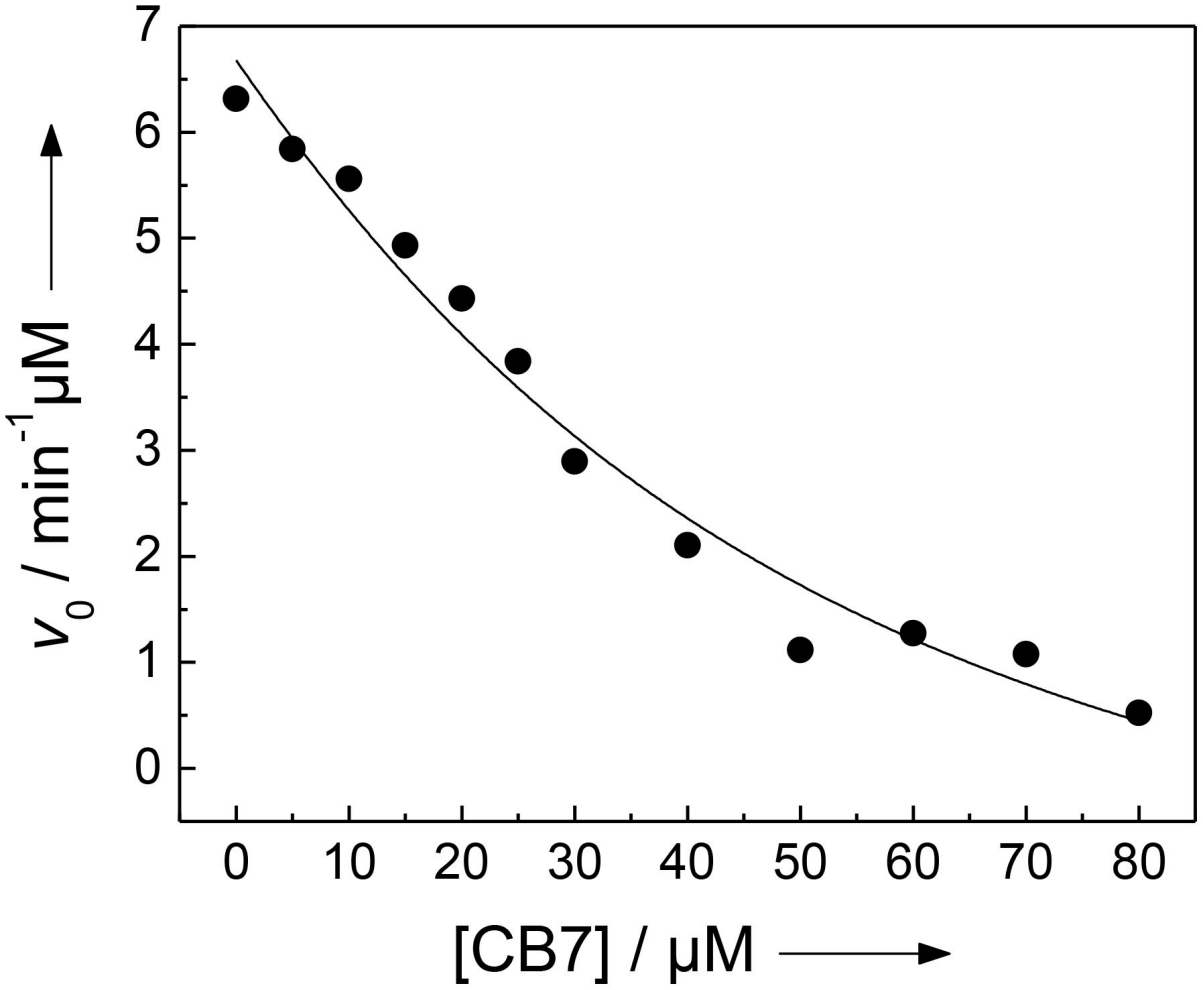
Inhibition of the activity of 300 nm trypsin against 30 μm H-(NO_2_)Tyr-Gly-Ser-Gly-Phe-Arg-Gly-Dbo-NH_2_ by CB7 (varying concentrations) in 112 mm borate buffer, pH 8.0. The solid line represents the fitted curve according to a 1:1 host-guest binding model.

We and others have previously described that CB7 can inhibit the activity of certain proteases by complexation of the substrate, which results in a “masking” of the enzyme recognition site in the substrate (Hennig et al., 2007c; Ghale et al., 2012; Logsdon and Urbach, 2013; Lee et al., 2015a). Conversely, the inhibition can be exploited to determine the binding constant with the enzyme recognition site of the protease substrate (McGarraghy and Darcy, 2000; Hennig et al., 2007c). The uncomplexed peptide H-(NO_2_)Tyr-Gly-Ser-Gly-Phe-Arg-Gly-Dbo-NH_2_ is a good substrate for trypsin having a catalytic turnover number of *k*_cat_ = 188 min^−1^ and a Michaelis-Menten constant of *K*_M_ = 229 μm (determined according to a Lineweaver-Burk plot, *n* = 7, *r* > 0.989). However, in the presence of varying amounts of CB7, the initial cleavage rate, *v*_0_, decreases below the detection limit (Figure 4) (Hennig et al., 2007c). A fitting according to a 1:1 binding model provided a binding constant of (3.2 ± 1.7) × 10^4^ m^−1^, which falls nicely in the range determined for the cleaved peptide and the strong binding constant from the UV absorption titration (*vide supra*). However, note that the recognition site for trypsin is the arginine side chain, while the complexation site of CB7 is the Dbo side chain. This seeming incongruity will be explained by the comprehensive binding model in the Discussion section.

### Enzyme Kinetics With Chymotrypsin

**Figure 5:**
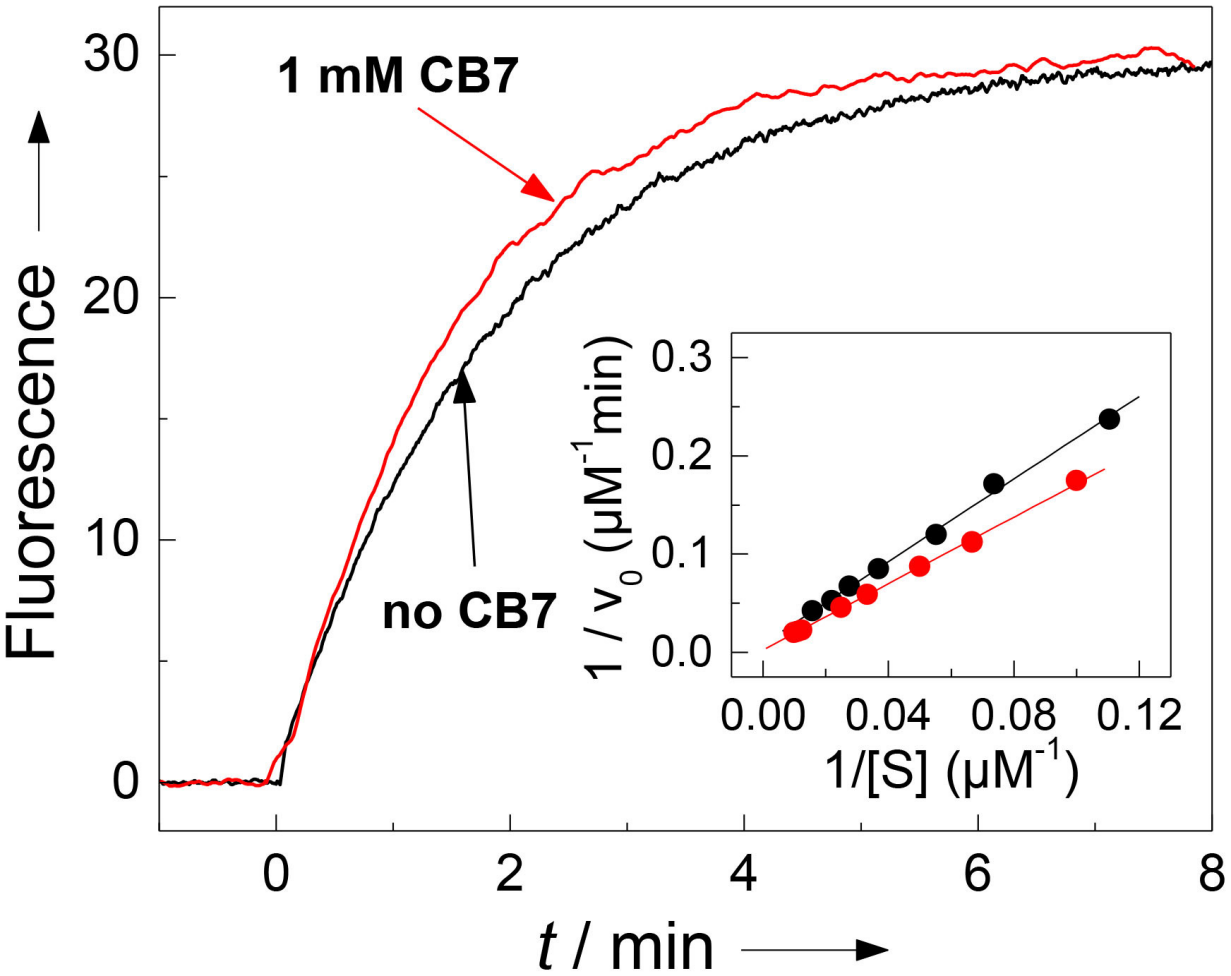
Influence of complexation of 30 μM H-(NO_2_)Tyr-Gly-Ser-Gly-Phe-Arg-Gly-Dbo-NH_2_ by CB7 on enzyme kinetics. The pseudo first-order rate constant *k*_cat_/*K*_M_ for cleavage by chymotrypsin (3 μM) is not significantly affected by complexation as can be seen from the fluorescence traces without CB7 (slower black trace, *k*_cat_/*K*_M_ = 1.90 μM^−1^min^−1^) and in the presence of 1 mM CB7 (faster red trace, *k*_cat_/*K*_M_ = 2.36 μM^−1^min^−1^). The Lineweaver-Burk plot without CB7 (black circles) and in presence of 1 mM CB7 (red circles) reveals the influence on the catalytic turnover number *k*_cat_ and on the Michaelis-Menten constant *K*_M_. The experiments were performed in 67 mM phosphate buffer, pH 7.0.

The results with chymotrypsin are clear-cut and show potential for application to Nano-TRF assays (*vide infra*). The pseudo first-order rate constant (*k*_cat_/*K*_M_), which is relevant at such low concentrations also used in many screening applications, is not much influenced by CB7 (1.90 min^−1^μm^−1^ in the absence and 2.36 min^−1^μm^−1^ in presence of 1 mm CB7). A more detailed investigation by Lineweaver-Burk plots (see Figure 5), however, revealed, that the CB7 complexation influences the kinetic properties in such a way that the influence on *k*_cat_ and *K*_M_ nearly compensate each other. In the absence of CB7, the peptide is cleaved with *k*_cat_ = 479 min^−1^ and *K*_M_ = 252 μm (*n* = 9, *r* > 0.999). Under conditions of complete complexation, i.e., in the presence of 1 mM CB7, the kinetic parameters were *k*_cat_ = 1,780 min^−1^ and *K*_M_ = 755 μm (*n* = 7, *r* > 0.998). This provides another independent evidence for the complexation of the peptide substrate and indicates that the transition state of the complex is more efficiently bound at the expense of the complexed substrate; in particular it suggests that the complexation takes place at other positions than at the phenylalanine residue, which is the recognition site for chymotrypsin. This finding is in accordance with a report pointing to a much lower binding affinity of phenylalanine residues within a peptide chain than at the *N*-terminal position (Urbach and Ramalingam, 2011).

### Model for the Substrate-CB7 Binding

According to the combined results from steady-state and time-resolved fluorescence spectroscopy as well as absorption spectroscopy and from the enzyme kinetic measurements it became evident that CB7 forms a 2:1 host-guest complex with the peptide substrate. The prolonged fluorescence lifetime confirms that binding of the Dbo side chain is strongest and that Dbo occupies the inner cavity of CB7. The second binding takes place at the (NO_2_)Tyr side chain, which can be most conveniently seen from the effects on the absorption spectrum.

**Figure 6:**
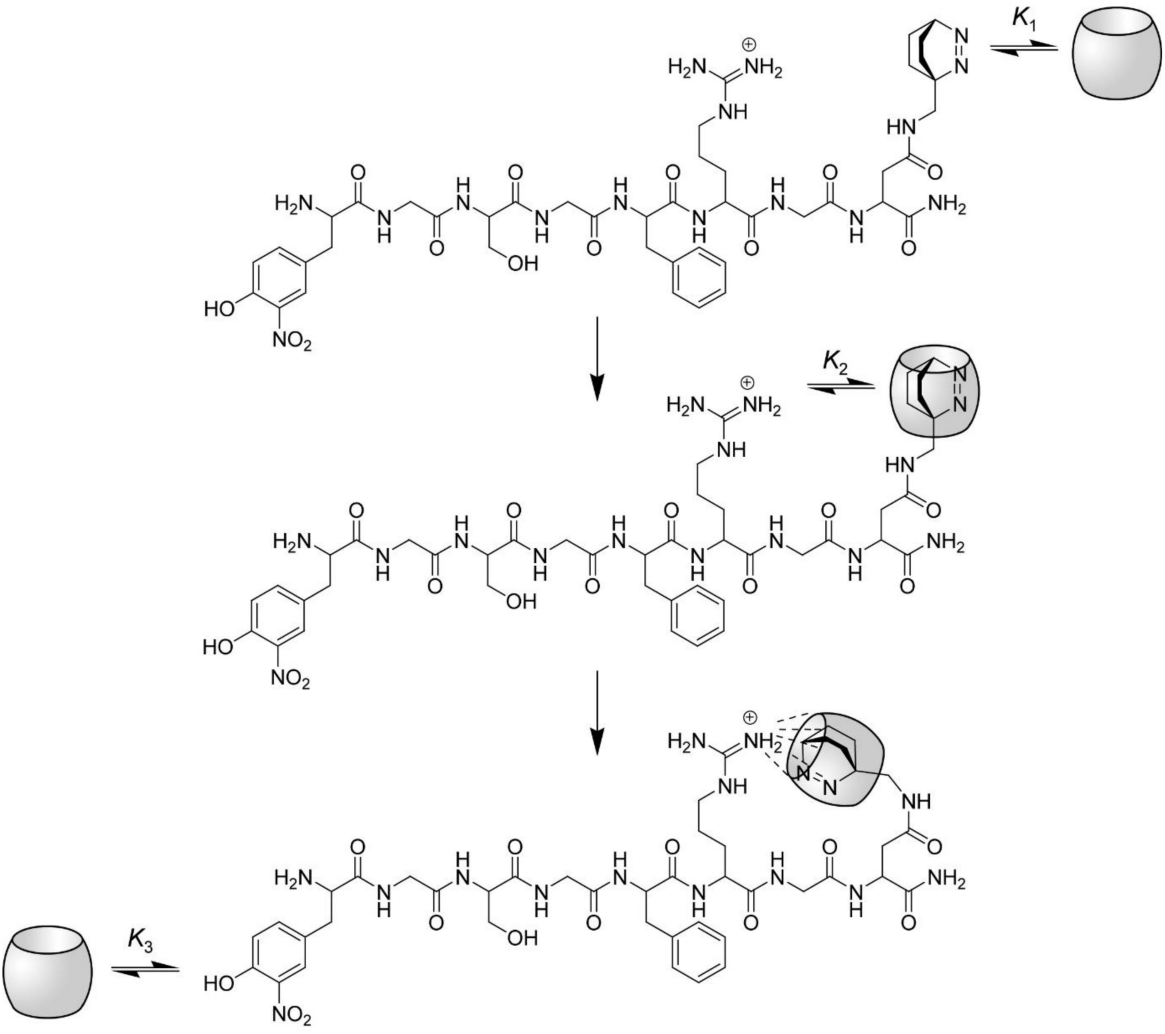
Proposed interactions between CB7 and the protease substrate H-(NO_2_)Tyr-Gly-Ser-Gly-Phe-Arg-Gly-Dbo-NH_2_. The complexation of the substrate by CB7 proceeds in three steps. At low CB7 concentration, the substrate is selectively complexed at the *C*-terminal Dbo residue with a binding constant *K*_1_. The upper rim of CB7 enables the complexation of small cationic residues (compare structure), although the inner cavity is already occupied. Intramolecular host-guest complexation with the arginine side chain takes thus place in the second step with a binding constant *K*_2_, which also provides an efficient masking of the arginine side chain against enzymatic cleavage by trypsin. Note that this mode of intramolecular binding is highly preferred (see text). Finally, at high CB7 concentrations the (NO_2_)Tyr residue is also complexed with a binding constant *K*_3_.

However, one problem still persists: How can it be that complexation of the Dbo residue leads to an inhibition of the arginine-recognizing enzyme trypsin, but not of the phenylalanine-recognizing chymotrypsin? We presume that the complexation properties of CB7 induce the formation of a “bidentate” inclusion/exclusion host-guest complex, in which DBO resides in the inner cavity of CB7 and the guanidinium group of the arginine side chain is bound to the remaining free portal (see Figure 6). NMR observations on other substrates have already suggested portal binding for the guanidinium side chain of an arginine residue and cavity binding of a hydrophobic residue (Hennig et al., 2007c). This model now also provides an excellent explanation for the seemingly different inhibition behavior of the substrate reported in this paper [inhibition as expressed by the binding constant is (3.2 ± 1.7) × 10^4^ m^−1^] and the previously communicated substrate *N*_α_-benzoyl-l-arginine *p*-nitroanilide [inhibition as expressed by the binding constant was (0.38 ± 0.06) × 10^4^ m^−1^]. The different inhibition of the activity of trypsin against those substrates does therefore not reflect the binding toward the arginine side chain, but reflects the binding of the hydrophobic residues and the subsequent intramolecular cyclization as proposed in Figure 6.

According to the Gaussian chain model, which relates the intermolecular binding constant *K*_inter_ to the intramolecular binding constant *K*_intra_ (Nau et al., 2003), the binding constants can be reduced to the ratio of their collision rate constants ($k_{\text{coll}}^{\text{inter}}$ and $k_{\text{coll}}^{\text{intra}}$) by assuming that the dissociation rate constant is not significantly affected by the intramolecular chain connecting the two chain ends. It follows that

(1) $$\begin{array}{l} \frac{K_{intra}}{K_{inter}}=\frac{k_{coll}^{intra}}{k_{coll}^{inter}}=\frac{1}{N_{A}}{\left(\frac{3}{2\pi Nb^{2}}\right)}^{\frac{3}{2}}\;for\;N\gg 1 \end{array}$$

with *N* representing the number and *b* representing the length of each chain segment. Assuming *N* = 15 (the number of connecting bonds) and an average bond length of *b* = 1.5 Å (Lide, 2003) one obtains for the ratio of *K*_intra_/*K*_inter_ = 3 M^−1^, where *K*_intra_ represents the ratio of the concentrations of the intramolecularly complexed vs. the uncomplexed form. This rough estimation demonstrates that there is no significant hindrance toward intramolecular complexation. Additional NMR studies with methylguanidinium (MeGua) chloride suggest that the formation of a ternary complex with CB7, DBO, and MeGua is indeed possible.

### Nano-TRF Assays

With the knowledge of the binding preferences of CB7 toward the protease substrate, it was possible to select a CB7 concentration, at which the fluorescent probe Dbo is selectively complexed. Trypsin is efficiently inhibited under this condition such that the feasibility for a continuous measurement of the enzymatic activity is sacrificed. However, the possibility for a stopped assay is not affected, i.e., the assay can still be carried out in the absence of CB7, CB7 is then added at known intervals after enzyme addition and the progress curves is derived from these measurements. Interestingly, CB7 also acts as the stopping reagent in this case, which remedies the necessity to perform, e.g., pH-jumps to stop the assay. In contrast, the minor influence on the activity of chymotrypsin is not deteriorating for a continuous measurement for this enzyme.

The steady-state intensity (expressed as the fluorescence quantum yield) of the Dbo fluorescence is slightly decreased inside CB7 (Mohanty and Nau, 2004), while the fluorescence lifetime is increased by ca. 3 times. The enhancement of adding CB7 in a Nano-TRF measurement is thus dependent on the applied delay time; at short delay times, the Nano-TRF intensity is smaller than the steady-state intensity, while at long delay times the opposite applies. In general, the Nano-TRF intensity can be theoretically predicted according to Equation (2), which relates two Nano-TRF intensities (*I*_1_ and *I*_2_) to their respective fluorescence lifetimes (τ_1_ and τ_2_) at different instrumental settings of the delay time, *t*_delay_, and gate time, *t*_gate_.

(2) $$\begin{array}{l} \frac{I_{1}}{I_{2}}=\frac{\int_{t_{\text{delay}}}^{t_{\text{delay}}+t_{\text{gate}}}A_{1}\cdot \;exp\left(-\frac{t}{\tau_{1}}\right)dt}{\int_{t_{\text{delay}}}^{t_{\text{delay}}+t_{\text{gate}}}A_{2}\cdot \;exp\left(-\frac{t}{\tau_{2}}\right)dt} \end{array}$$

**Figure 7:**
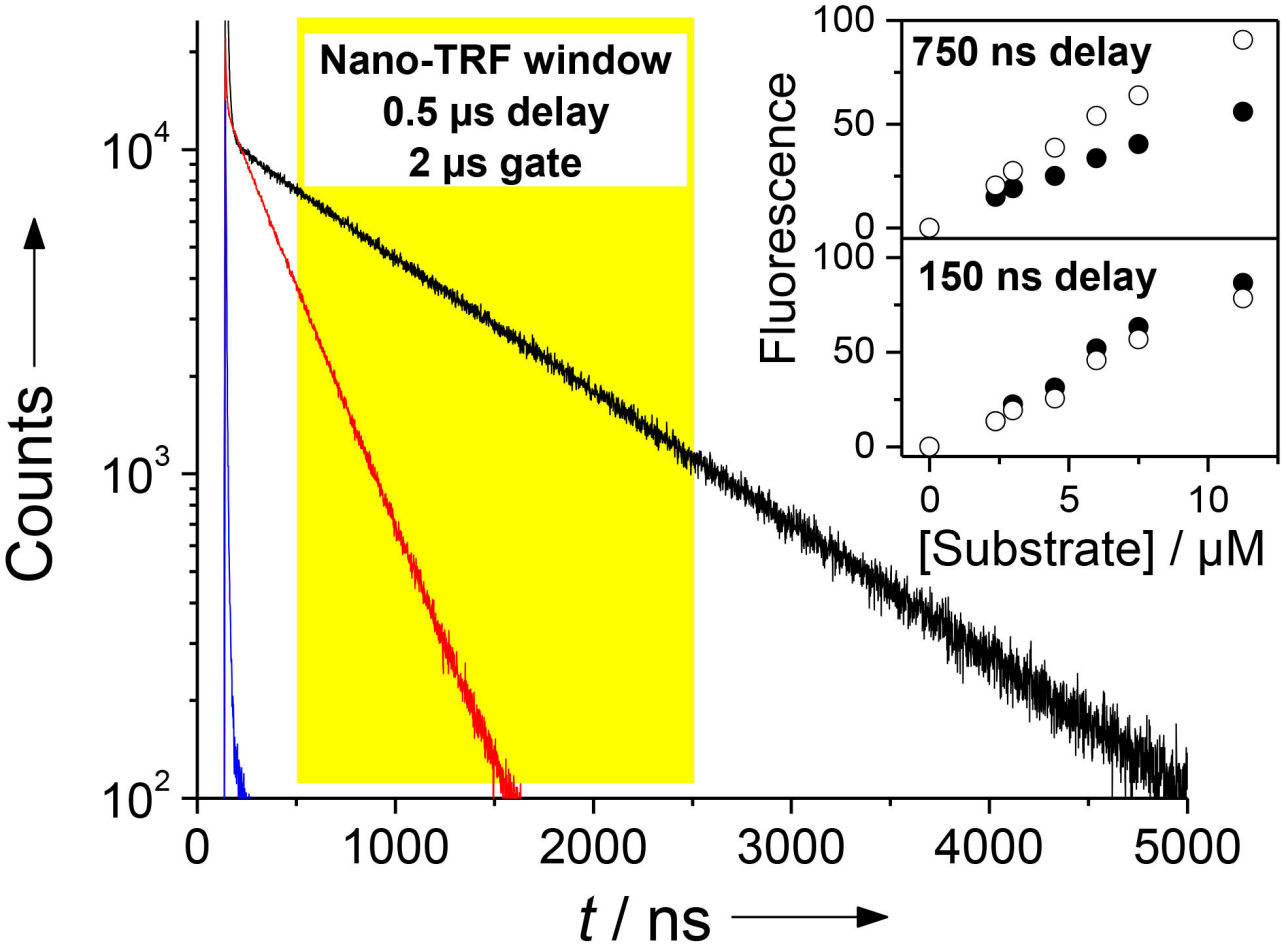
Demonstration of the Nano-TRF principle for a CB7-enhanced assay. Shown are the fluorescence decay traces for the uncleaved peptide (τ ≈ 5 ns, blue), the peptide after proteolytic cleavage in the absence of CB7 (τ = 325 ns, red), and after proteolytic cleavage in the presence of 100 μm CB7 (τ = 950 ns, black). The assay was performed with 30 μm H-(NO_2_)Tyr-Gly-Ser-Gly-Phe-Arg-Gly-Dbo-NH_2_ in 67 mm phosphate buffer, pH 7. Detection in the Nano-TRF mode is indicated by the yellow box (set to a delay time of 500 ns). The prolonged fluorescence lifetime translates directly into an enhanced fluorescence intensity in Nano-TRF measurements at delay times >150 ns, which is shown in the inset for delay times of 150 and 750 ns (solid circles without CB7, open circles with 100 μm CB7). Note that our instrumentation unfortunately only offered a gate time of 2 μs.

Considering the fluorescence quantum yields of Dbo (0.26) and the Dbo/CB7 complex (0.19) (Mohanty and Nau, 2004) and their respective fluorescence lifetimes (325 and 950 ns) one obtains a ratio of preexponential factors of *A*_Dbo/CB7_/*A*_Dbo_ of 0.25. A calculation with these values affords that the Nano-TRF intensity of Dbo and the Dbo/CB7 complex are equal for *t*_delay_ ≈ 200 ns and *t*_gate_ = 2 μs. With *t*_delay_ > 200 ns a higher Nano-TRF intensity is expected for the Dbo/CB7 complex, which was experimentally confirmed in a convincing manner (Figure 7). In this context, it is important to note that usually delay times of 150 ns are sufficient to suppress the short-lived background fluorescence, but longer delay times are beneficial to enhance the differentiation of substrate and product (Johansson et al., 2004; Hennig et al., 2007c).

In conclusion, we have established herein CB7 as a supramolecular additive to enhance the sensitivity Nano-TRF assays. A peptide substrate for trypsin and chymotrypsin was doubly labeled with Dbo as a fluorescent probe and 3-nitrotyrosine as a FRET-based quencher. CB7 binds to the Dbo residue in the enzyme substrate, which has only a small effect on the fluorescence lifetime, whereas the fluorescence lifetime of the cleaved product increased from 325 to 950 ns. This facilitates the discrimination of substrates and products. While the presence of CB7 had only a small effect on the enzyme kinetics with chymotrypsin, the activity of trypsin was nearly completely inhibited, which suggested a hairpin conformation of the peptide, in which the guanidinium side chain of the arginine residue is additionally bound to a vacant ureido rim of one of the CB7 hosts.

## Data Availability Statement

All datasets generated for this study are included in the article/Supplementary Material.

## Author Contributions

AH and WN jointly conceived the study, interpreted the results, and wrote the manuscript. The experiments were performed by AH. All authors contributed to the article and approved the submitted version.

## Conflict of Interest

The authors declare that this study received funding from F. Hoffmann-La Roche Ltd. The funder was not involved in the study design, collection, analysis, data interpretation, and writing of this article or the decision to submit it for publication.

## Footnotes

^1^*k*_diff_ is determined from the Smoluchowski equation: *k*_diff_ = 4π*N*_A_(*r*_A_ + *r*_B_)(*D*_A_ + *D*_B_). The diffusion coefficient for 3-nitrotyrosine (6.30 × 10^−6^ cm^2^ s^−1^) was determined according an empirical formula for amino acids, peptides and proteins (*D* = 10^−7^) exp(α – β ln *M*, where α = 6.42, β = 0.42, and *M* is the molecular weight). The diffusion coefficients of DBO and the CB7/DBO complex were determined according to the Stokes-Einstein relation *D* = (*kT*)/(6π*r*η). The molecular shape of DBO is approximated by a sphere with a radius *r* = 2.3 Å. CB7 can be best described as an oblate spheroid with *r*_a_ = 8.0 Å and *r*_b_ = 4.55 Å, which gives an effective radius of *r* = ($r_{\text{a}}^{2}$*r*_b_)^1/3^ = 6.6 Å. The diffusion coefficients are thus 9.32 × 10^−6^ cm^2^ s^−1^ for DBO and 3.25 × 10^−6^ cm^2^ s^−1^ for the CB7/DBO complex. The reaction radius (*r*_A_ + *r*_B_) was taken as 4 Å = 4 × 10^−8^ cm [see (Stryer and Haugland, 1967; Yokota and Tanimoto, 1967; Steinberg and Katchalski, 1968; Thomas et al., 1978) for details].

^2^Taking the reduced mutual diffusion coefficient of biopolymer chain ends into account (see Wang et al., 2003).

^3^Note that the extinction coefficient of Dbo is ca. 50times smaller than the extinction coefficient of (NO_2_)Tyr; the absorption of Dbo is thus virtually invisible and can be neglected.
