# Supplementary material for: Interaction of Cucurbit[7]uril With Protease Substrates: Application to Nanosecond Time-Resolved Fluorescence Assays
Source: Front Chem. 2020 Sep 10;8:806. doi: 10.3389/fchem.2020.00806 (PMC7511663; doi:10.3389/fchem.2020.00806)
Supplement: Supplementary file 1 [file Data_Sheet_1.PDF]

SUPPORTING INFORMATION FOR

Interaction of Cucurbit[7]uril with Protease Substrates: Application to Nanosecond Time-Resolved Fluorescence Assays

Andreas Hennig[1,2,3*], Werner M. Nau[1]

[1] Department of Life Sciences and Chemistry, Jacobs University Bremen gGmbH, Bremen, Germany

[2] Institute of Chemistry of New Materials, School of Biology/Chemistry, Universität Osnabrück, Osnabrück, Germany

[3] Center of Cellular Nanoanalytics (CellNanOs), Universität Osnabrück, Osnabrück, Germany

andreas.hennig@uni-osnabrueck.de

Representative Calculation of Förster Radius

The efficiency of fluorescence resonance energy transfer E_T can be determined by the ratio of fluorescence intensities in presence and absence of the acceptor F_{da}/F_d.[S1] Furthermore, E_T is, according to Förster's theory, dependent on the distance R between donor and acceptor and the Förster radius R_0 (equation S1):

$$E_T = 1 - \frac{F_{da}}{F_d} = \frac{R_0^6}{R_0^6 + R^6} \qquad [S1]$$

R_0 is the distance at which one half of the excitation energy is transferred from the donor to the acceptor and is determined by the spectroscopic and physical properties of the donor and acceptor as well as their environment. R_0 can be calculated by equation S2

$$R_0 = \sqrt[6]{8.79 \times 10^{-5} \kappa^2 n^{-4} Q_d J(\lambda)} \qquad [S2]$$

in which κ^2 is the orientation factor of the transition dipole moments of the donor and the acceptor, n is the refractive index of the surrounding medium, Q_d is the fluorescence quantum yield of the donor in the absence of the acceptor, and $J(\lambda)$ is the overlap integral. $J(\lambda)$ is calculated by equation S3.

$$J(\lambda) = \frac{\int F_d(\lambda)\varepsilon(\lambda)\lambda^4 d\lambda}{\int F_d(\lambda) d\lambda} \qquad [S3]$$

in which F_d is the fluorescence intensity of the donor, ε is the extinction coefficient of the acceptor with λ as integration parameter. The unit of $J(\lambda)$ is typically given as $M^{-1}cm^{-1}nm^4$.

To calculate $J(\lambda)$, corrected fluorescence spectra of DBO and absorption spectra of nitrotyrosine at the respective pH were measured. The fluorescence spectra were integrated between 360-650 nm using OriginPro 2016 to afford the denominator in equation S3 and the product of the fluorescence intensity values, the extinction coefficient, and the wavelength to the power of four were integrated between 360-650 nm to afford the numerator in equation S3.

The orientation factor κ^2 is commonly taken as 2/3 for a system in which rapid three-dimensional averaging of the mutual orientation of transition dipole moments within the excited-state lifetime of the donor occurs,[S1] which is a valid approximation for terminally labelled peptides, and the refractive index was taken as 1.333 (refractive index of water).[S1-S3] Using equation S2 gives the Förster, which is then used in combination with equation S1 to give the energy transfer efficiencies in Table 1 in the main text.

(S1) Lakowicz, J. R. *Principles of Fluorescence Spectroscopy*; 3rd ed.; Springer: New York, 2006.

(S2) Sahoo, H.; Roccatano, D.; Zacharias, M.; Nau, W. M.; "Distance Distributions of Short Polypeptides Recovered by Fluorescence Resonance Energy Transfer in the 10 .ANG. Domain", *J. Am. Chem. Soc.* **2006**, *128*, 8118.

(S3) Sloniec, J.; Schnurr, M.; Witte, C.; Resch-Genger, U.; Schröder, L.; Hennig, A.; "Biomembrane Interactions of Functionalized Cryptophane-A: Combined Fluorescence and 129Xe NMR Studies of a Bimodal Contrast Agent", *Chem. Eur. J.* **2013**, *19*, 3110.
